# MetaboLink: a web application for streamlined processing and analysis of large-scale untargeted metabolomics data

**DOI:** 10.1093/bioinformatics/btae459

**Published:** 2024-07-17

**Authors:** Ana Mendes, Jesper Foged Havelund, Jonas Lemvig, Veit Schwämmle, Nils J Færgeman

**Affiliations:** Department of Biochemistry and Molecular Biology, University of Southern Denmark, 5230 Odense M, Denmark; Department of Biochemistry and Molecular Biology, University of Southern Denmark, 5230 Odense M, Denmark; Department of Biochemistry and Molecular Biology, University of Southern Denmark, 5230 Odense M, Denmark; Department of Biochemistry and Molecular Biology, University of Southern Denmark, 5230 Odense M, Denmark; Department of Biochemistry and Molecular Biology, University of Southern Denmark, 5230 Odense M, Denmark

## Abstract

**Motivation:**

The post-processing and analysis of large-scale untargeted metabolomics data face significant challenges due to the intricate nature of correction, filtration, imputation, and normalization steps. Manual execution across various applications often leads to inefficiencies, human-induced errors, and inconsistencies within the workflow.

**Results:**

Addressing these issues, we introduce MetaboLink, a novel web application designed to process LC-MS metabolomics datasets combining established methodologies and offering flexibility and ease of implementation. It offers visualization options for data interpretation, an interface for statistical testing, and integration with PolySTest for further tests and with VSClust for clustering analysis.

**Availability and implementation:**

Fully functional tool is publicly available at https://computproteomics.bmb.sdu.dk/Metabolomics/. The source code is available at https://github.com/anitamnd/MetaboLink and a detailed description of the app can be found at https://github.com/anitamnd/MetaboLink/wiki. A tutorial video can be found at https://youtu.be/ZM6j10S6Z8Q.

## 1 Introduction

Untargeted metabolomics approaches are generally hypothesis-generating focusing on annotating metabolites and monitoring metabolic changes under different conditions to identify affected pathways (Schrimpe-Rutledge *et al.* 2016). Metabolites are typically detected by specific detection techniques including nuclear magnetic resonance (NMR) spectroscopy and liquid chromatography-mass spectrometry (LC-MS). Raw signals are then pre-processed to produce data in a suitable format for subsequent statistical analysis. The metabolites are identified based on spectral information using appropriate databases. Post-processing methods and statistical analysis are used to identify significantly expressed metabolites in different samples, groups, or conditions. The significantly expressed metabolites are linked to the biological context by using enrichment and pathway analysis (Data interpretation). This data can finally be integrated with other omics data to gain a comprehensive understanding of the molecular mechanisms (Chen *et al.* 2022).

## 2 Materials and methods

MetaboLink was developed in R and runs as a web application on a Shiny server. Common normalization methods such as normalizing by the total sum, or the median of the sample are available in MetaboLink. At the same time, different types of regression methods can be used to find the drift pattern and normalize the data. For instance, Locally Estimated Scatterplot Smoothing (LOESS) is used in drift correction to correct for peak area drift based on the observed intensity drift in quality control (QC) samples (Dunn *et al.* 2011). An alternative normalization tool available is the Probabilistic Quotient Normalization (PQN). PQN is based on the calculation of the most probable dilution factor (Dieterle *et al.* 2006). This calculation is based on the distribution of the quotients of each sample’s spectrum by those of a reference spectrum, e.g. the QC samples.

MetaboLink offers common imputation methods, such as using the mean or the median of observed values. It also incorporates the classification algorithms K-nearest Neighbors (kNN) and Random Forest (RF) to try to find the best value to impute. However, imputation should be used only if strictly necessary and the app offers the option to only impute QC samples for methods requiring full coverage of these. PolySTest (Schwämmle *et al.* 2020) and VSClust (Schwammle and Jensen 2018) do not require full coverage.

Differential Analysis with Limma takes advantage of the respective R package (Ritchie *et al.* 2015) for statistical analysis, facilitating the comparison between two groups, analysis against a reference group, and evaluation of groups with paired samples. To control the false discovery rate, correction for multiple testing using Benjamini–Hochberg method (Benjamini and Hochberg 1995) is applied to all tests. PolySTest is also integrated into the workflow of MetaboLink. By exporting the dataset to PolySTest, it is possible to conduct statistical analysis using the “miss test” method. This method tackles the challenge of reduced feature coverage resulting from missing values and results in higher confidence and enhanced sensitivity.

## 3 Results

The web application MetaboLink was designed such that researchers without particular computational knowledge also can take advantage of computational methods to gain insights into the biological meaning of metabolomics and lipidomics datasets.

**Figure 1.**
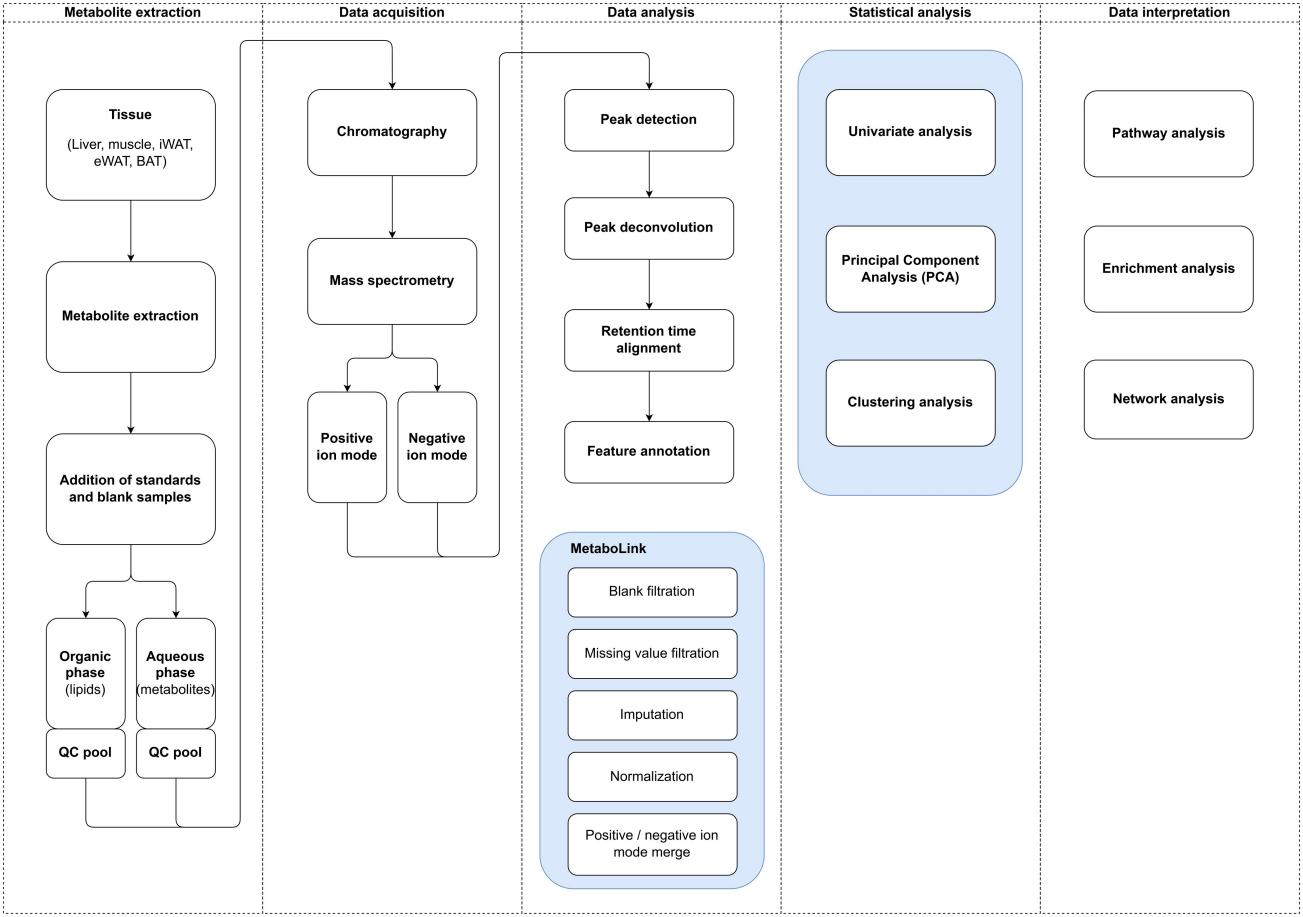
Overview of untargeted metabolomics/lipidomics workflows and how MetaboLink (marked) can be used to process metabolomics and lipidomics datasets.

Users can upload intensity measurements and experiment metadata from untargeted LC-MS experiments through the graphical interface. The app permits adjusting the metadata file to prevent errors and supports data file modifications, such as sample removal and renaming. Features include data post-processing (imputation, missing value and blank filtration, normalization, log-transformation, scaling), merging datasets acquired with different ion modes, and conducting statistical tests for various experimental designs (Fig. 1).

Users can export the datasets directly to PolySTest and VSClust to perform statistical tests and clustering, respectively. A formatted dataset compatible with the MetaboAnalyst (Pang *et al.* 2024) software can also be downloaded.

MetaboLink also incorporates visualization tools tailored for metabolomics and lipidomics data analysis. The application offers various plots to visualize the median values across samples and quality controls, enhancing data interpretation. Principal component analysis (PCA) allows comparing datasets (e.g. pre- and post-normalization) within PCA panel. In addition, MetaboLink provides functionalities for viewing feature drift and includes a feature viewer for comparison of specific features across different groups.

## 4 Discussion

MetaboLink provides a data analysis protocol specific for metabolomics and lipidomics research. It addresses the demand for user-friendly data post-processing by integrating a comprehensive set of analytical tools in a user-friendly interface. We recommend to carefully navigate through the post-processing steps and evaluate at each stage to make sure that the chosen methods align with the specific dataset being analyzed.

MetaboLink not only provides an accessible platform for researchers regardless of their computational expertise but also offers seamless integration with further analysis platforms and visualization tools such as MetaboAnalyst.
